# Guided Endodontics for a Tooth with Root Fracture: A Case Report

**DOI:** 10.3390/jcm14124079

**Published:** 2025-06-09

**Authors:** Monika Kuczmaja, Agata Żółtowska

**Affiliations:** Department of Conservative Dentistry, Faculty of Medicine, Medical University of Gdańsk, 80-210 Gdańsk, Poland; monika.kuczmaja@gumed.edu.pl

**Keywords:** guided endodontics, CBCT, root canal treatment, 3D-printed template, pulp canal obliteration

## Abstract

**Background:** A 19-year-old female patient reported to the Department of Conservative Dentistry, Medical University of Gdańsk, with pain in the left lateral incisor. During the attempt to perform root canal treatment on tooth 22, we encountered difficulties in locating the canal opening, which resulted in treatment failure. Radiographic examinations revealed Pulp Canal Obliteration and a root fracture with a double fracture line, resulting in two root fragments. The prognosis for this tooth was poor, with extraction being the most likely outcome. **Methods:** To provide effective therapy, a 3D-printed endodontic guide was utilized, allowing for more precise localization of the canal in a tooth with a calcified root canal and complex fracture morphology. An intraoral scan and cone-beam computed tomography (CBCT) were performed, followed by the design of the guide. This facilitated accurate planning of the entry path for endodontic instruments, promoting minimally invasive treatment and reducing the loss of tooth structure. **Results:** Through navigated endodontics, the treatment became more predictable, and the risk of iatrogenic complications was minimized, significantly improving the prognosis of the therapy. Clinical and radiological examinations conducted after 12 months demonstrated successful treatment and preservation of the tooth in the oral cavity. **Conclusions:** The obtained results suggest that the use of guided endodontics can improve outcomes in cases with pulp canal obliteration and complex fractures, offering a minimally invasive and predictable approach.

## 1. Introduction

Disorders in the oral cavity can impact speech, functionality, nutritional status, and appearance, leading to diminished social interactions and family life [1]. The loss of even a single tooth can disrupt the balance of the stomatognathic system, resulting in occlusal imbalances, tooth wear, and bruxism. Disruptions in the occlusal plane can result in temporomandibular joint neuralgia, headaches (migraines), and pain in surrounding muscles [2].

To reduce the incidence of tooth extractions, effective endodontic treatment or endodontic microsurgical procedures are essential. In certain clinical cases, it is impossible to perform root canal therapy, or it may be conditional. Such situations typically arise when the chamber or the root canal of the tooth is obliterated, meaning that its lumen is closed (Pulp Canal Obliteration, PCO). Obliteration may occur due to trauma [3], tooth luxation, orthodontic treatment, or the deposition of tertiary dentin with age.

In teeth with obliteration, searching for the canal opening results in a significant loss of the tooth’s own tissue. Excessive preparation, especially in the cervical area, may lead to subsequent tooth fractures and the inability to achieve a tight restorative or prosthetic reconstruction. Searching for a calcified root canal can be time-consuming and carries an increased risk of perforation [4] or creation of via falsa—false pathways. The process of obliteration formation is usually asymptomatic for the patient. PCO is most often detected incidentally during an X-ray examination or is noticeable due to a change in the color of the tooth. In some cases, three-dimensional imaging, such as CBCT, is necessary. CBCT is utilized in endodontics to assess complex root canal morphology, identify resorption and root fractures, and conduct preoperative evaluations for retreatment [5]. Other indications include determining missed or calcified root canals, differential diagnosis of periapical changes, and detecting early inflammatory changes at the root apex that are not visible on 2D radiographs [6,7].

**Figure 1:**
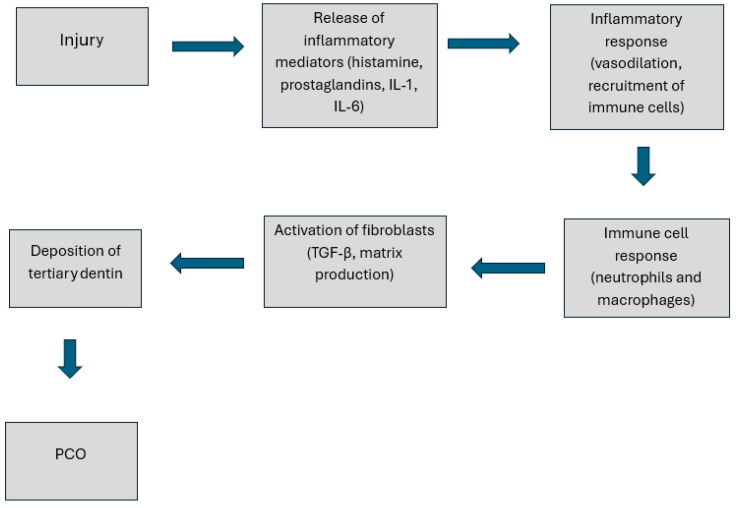
Pathophysiological mechanisms of Pulp Canal Obliteration: schematic of cellular responses and inflammatory processes.

PCO, also known as calcific metamorphosis, is a process characterized by the deposition of hard tissue, referred to as tertiary dentin, along the walls of the root canal [4]. It can lead to a reduction in the volume of the pulp space and the diameter of the root canal. Pulp Canal Obliteration is a common finding in young permanent teeth following trauma, especially luxation [8], with its prevalence estimated in the literature at 3.7–40% for all dental traumas and 29.4–95.2% after root fractures [4]. Studies by Holcomb and Gregory, Jacobsen, and Kerekes have shown that up to 16% of teeth with calcific metamorphosis ultimately develop pulpal necrosis [9,10]. PCO manifests itself as discoloration and a decreased response to vitality tests, which may require treatment to improve esthetics [11]. It is a reaction to pulp damage leading to tertiary dentin deposition and canal narrowing. Trauma initiates inflammation and tertiary dentin deposition by fibroblasts [12,13]. Age, previous treatment, and chronic inflammation also affect PCO. The inflammatory process at the cellular level is shown in Figure 1.

## 2. Materials and Methods

**Figure 2:**
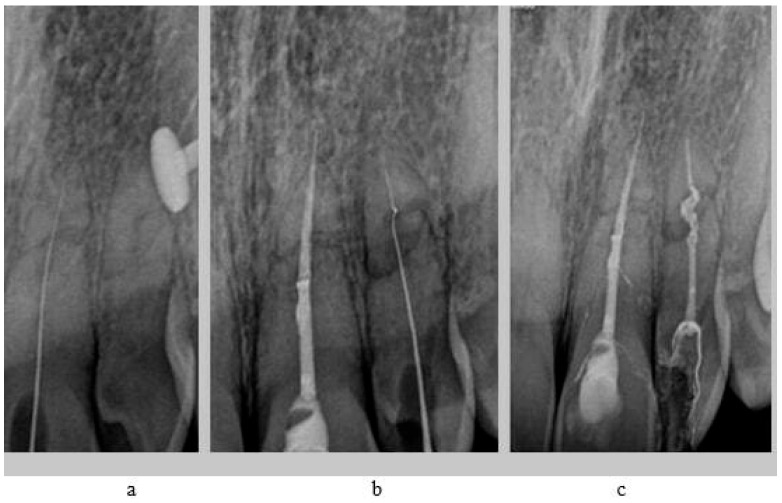
RVG of tooth 22 (**a**) before treatment, (**b**) during treatment (with instrument in canal), (**c**) after treatment (canal filled with gutta-percha and AH Plus).

A 19-year-old female patient reported to the Department of Conservative Dentistry Medical University of Gdańsk due to pain in tooth 22 (upper left lateral incisor, according to the Federation Dentaire Internationale (FDI) dental numbering system) and 21 (lateral left incisor) [14]. In her medical history, it was noted that she suffered an injury in 2021 after being hit by a car while riding her bicycle, which resulted in a two-week traumatic coma and a diagnosis of a traumatic brain injury. Following this incident, the patient was referred for outpatient treatment and dental consultation. During the dental examination, pathological mobility (Grade 2) (Miller’s index of mobility) [15] was observed in teeth 11, 21, and 22. Teeth 12, 21, and 22 appropriately responded to electrical stimuli, while tooth 11 showed no reaction to the Pulp tester (Yusendent, China). Radiographically, a Class VI injury according to the Ellis Classification [16] was identified in teeth 21 and 22, and a Class VII injury in teeth 12 and 11. The patient was informed about the necessity of follow-up visits; however, she did not attend. In April 2024, the patient returned complaining of pain in teeth 21 and 22. She described the pain as constant, occurring when biting, worsening with hot foods, and lasting at night. Clinical examination revealed pain upon vertical percussion and no reaction to electrical stimuli. The buccal mucosa of the alveolar process was found to be without clinical changes, and there was no tenderness when palpating the alveolar process. Radiovisiography (RVG) (Carestream 9300C, Carestream Dental, 2018, Atlanta, GA, USA) did not show any periapical changes. Notably, the RVG revealed a complex root fracture in tooth 22, characterized by a double fracture line and two distinct root fragments (Figure 2a), which is generally an indication of tooth extraction. However, due to the young age of the patient and the psychological aspect, it was decided to perform conditional endodontic treatment. The patient was informed about the possibility of treatment failure and the need for extraction. The patient accepted the presented treatment plan. During a pain emergency visit, an attempt was made to trepan teeth 21 and 22 under infiltration anesthesia with Dentocaine (40 mg/0.01 mg/1 mL, 1 ampoule, Inibsa, Madrid, Spain); however, the canal in tooth 22 was not locatable. A temporary filling (Kromoglass 2 (20 g) Lascod, Milano, Italy) was placed, and tooth 22 we classified for treatment utilizing an endodontic guide.

**Figure 3:**
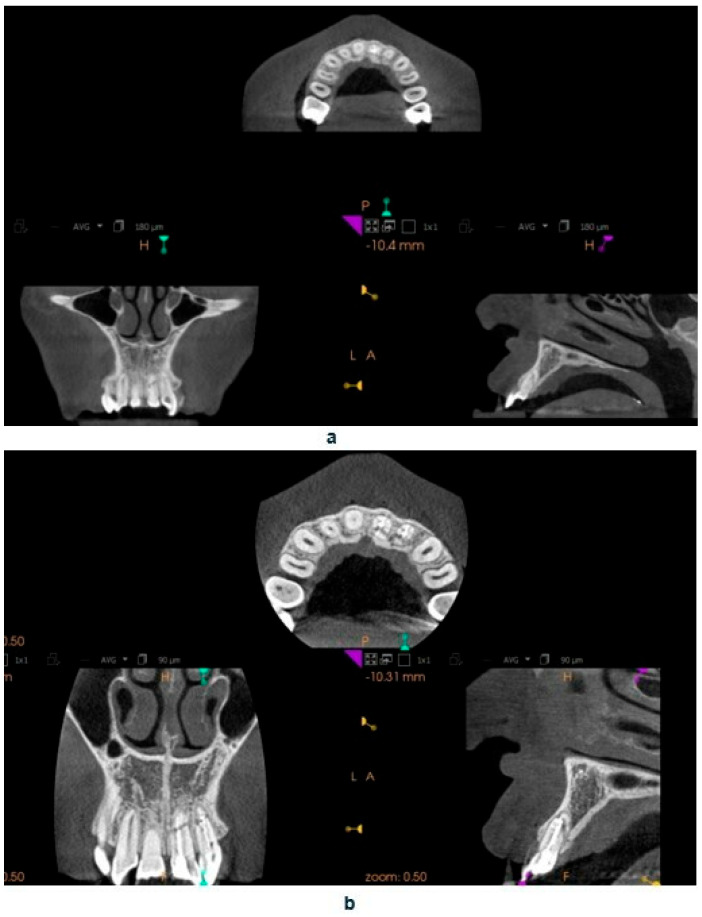
CBCT images presenting tooth no. 22: (**a**) before treatment, (**b**) after treatment.

In tooth 21, we successfully performed the root canal treatment with minimal loss of tooth tissue, covering both fragments of the fractured tooth root. The aim of the study is to assess whether the treatment of tooth 22 with root canal obliteration using endodontic navigation will bring positive clinical and radiological effects. The use of the endodontic template will be evaluated through clinical and radiological assessments of the tooth before the initiation of endodontic treatment and after the completion of the treatment. After obtaining both written and verbal consent to perform the endodontic procedure, an endodontic template was designed. The patient presented for root canal treatment of tooth 22. During the initial visit, a CBCT (Carestream 9300C, Carestream Dental, 2016, Atlanta, GA, USA) of the maxillary arch was completed (Figure 3a), and an intraoral scan (Carestream 3600) of the jaw was performed.

The standard tessellation language (STL) (a file format native to the stereolithography CAD software created by the 3D system) files containing the intraoral scan and the CBCT of the entire dental arch were uploaded into the Blue Sky Plan 4 software. Within this program, a template was designed that included a sleeve intended to guide the endodontic instrument, specifically the Munce Discovery Bur (CJM Engineering, Santa Barbara, CA, USA) used for locating the root canal opening.

**Figure 4:**
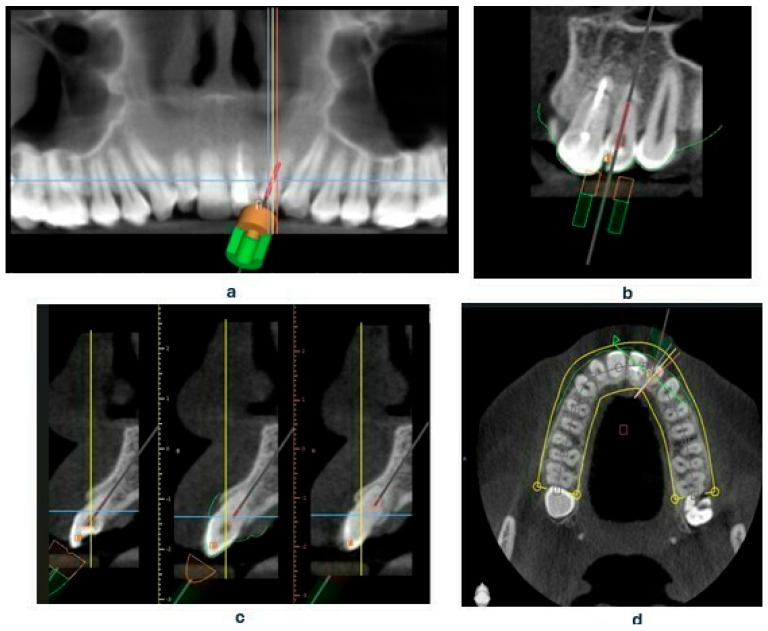
Presenting the creation of an endodontic template, (**a**) settings of drill position in Blue Sky Plan software panoramic view, (**b**) coronal view, (**c**) sagittal view, (**d**) axial view.

Based on the CBCT images and the scans, a template was designed that covers the surfaces of all teeth in the arch, including the palatal and labial surfaces [17]. The template is set to plan the drill position—including the position, length—0 mm, apical diameter—1.05 mm, occlusal diameter—1.55 mm, as well as the dimensions of the software guide tube (offset—10.5 mm—meaning the distance from the tooth, height—5 mm, and guide hole diameter—1.05 mm) (Figure 4a–d). Additionally, the planned length for accessing the root canal will also be determined—18 mm.

**Figure 5:**
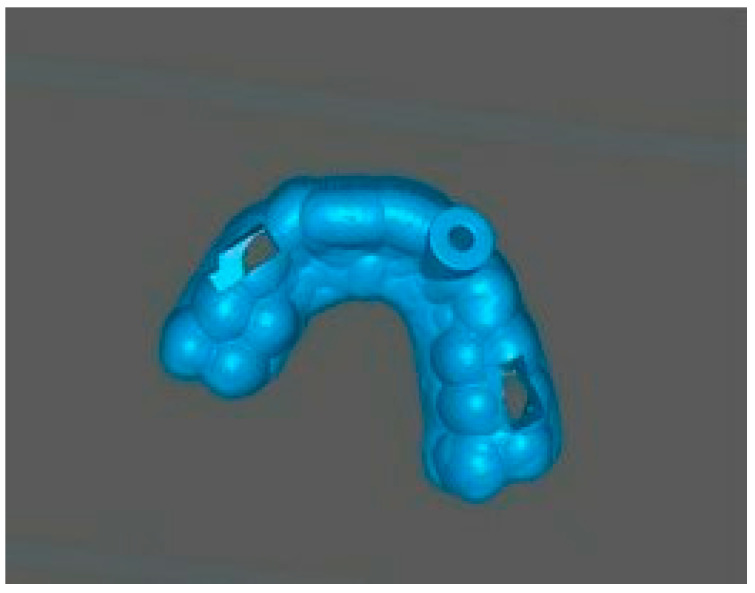
STL file of the guide imported to the slicer software (Chitubox version 2.0.0).

After the design was approved, the STL file of the template project was exported from the planning software (Blue Sky Plan 4 version 4.12.3) and processed in Chitubox software version 2.0.0 (Figure 5).

**Figure 6:**
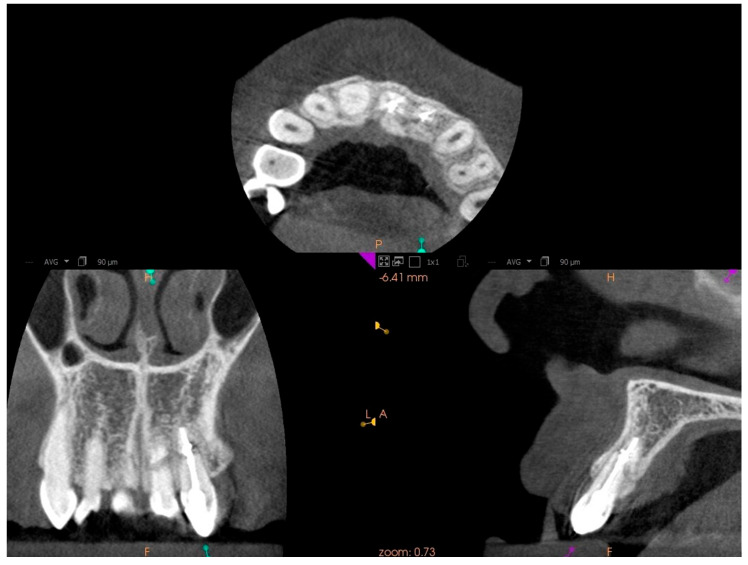
CBCT images presenting tooth no. 22 after 12 months of treatment.

**Figure 7:**
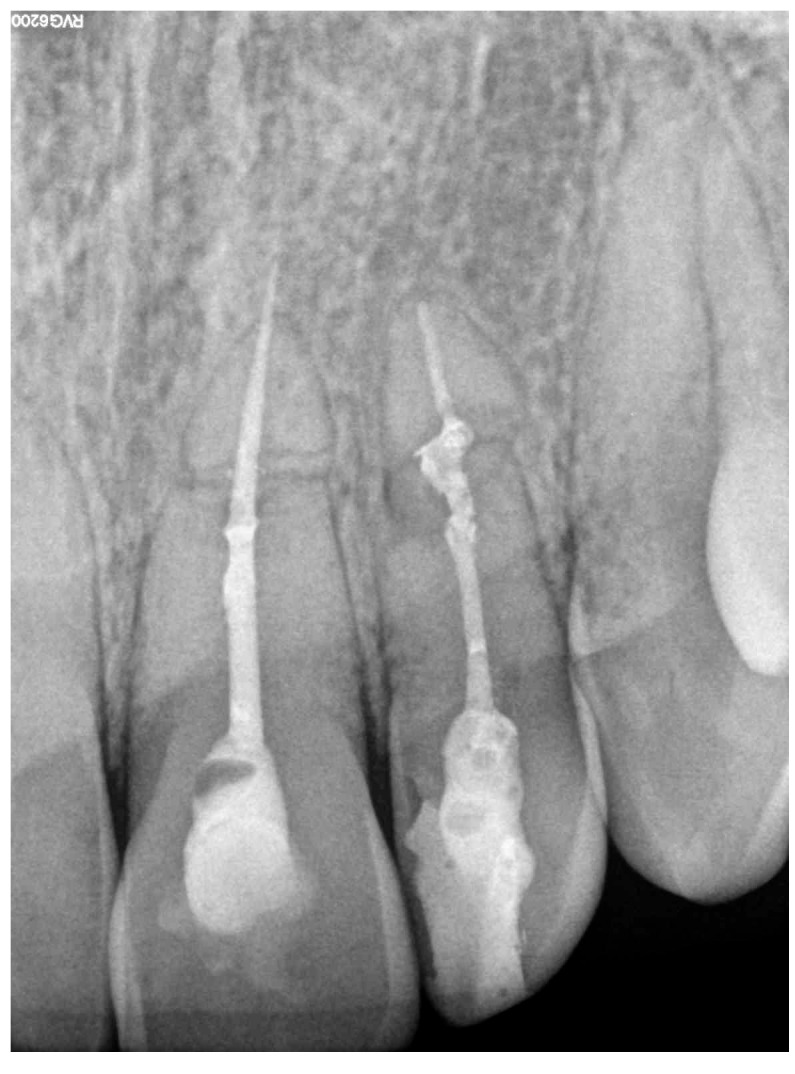
RVG of tooth 22 after 12 months of treatment.

This software allowed for the preparation of the file for 3D printing by adding supports and slicing the template into layers of 50 µm thickness. The template was then printed using dedicated transparent resin—Class IIa (NextDent SG, Istanbul, Turkey) on a 3D printer (Phrozen Sonic Mini 4K, Taipei, Taiwan) [18,19]. The post-processing phase was conducted in strict conformity with the resin manufacturer’s instructions to prevent dimensional alterations [20]. The placement of the template in the oral cavity was checked, and the treatment area was isolated. The patient was locally anesthetized with infiltration using Dentocaine (1 ampoule, Inibsa, Madrid, Spain). The temporary filling was removed from tooth 22. The template was placed on the upper arch, and canal access was initiated using a Munce bur #1 (Munce Discovery Bur^TM^). The template was removed after the bur reached a depth 2 mm greater than previously planned, to irrigate the root canal with a 5.25% NaOCl solution, allowing for endodontic access control and cleaning of the Munce bur. The work continued until the bur achieved the planned length previously set. The position of the canal was checked using C-pilots (VDV, Munich, Germany) ISO 10 and ISO 15. Following that, RVG was performed (Figure 2b) to confirm the correct position of the instrument in the canal and the length of the canal. The canal was prepared using Reciproc Blue (VDV, Munich, Germany) endodontic instruments to the working length, confirmed by measurements with an endometer (Raypex 5, VDW, Munich, Germany) and radiographically. The canal was irrigated using protocol along with ultrasonic activation (PUI) with 5.25% NaOCl, 40% citric acid, and triple-distilled water. After drying the root canal with a paper point (Reciproc 25, VDV), the canal was filled with gutta-percha by a Continous Wave Obturation (CWO) (Reciproc 25, VDV, and BeeFill 2in1 Obturation Kit, VDW GmBH, Munchen, Germany) with the sealer AH Plus (Dentsply DeTrey GmbH, Philadelphia, PA, USA). After filling the canal, RVG (Figure 2c) and CBCT imaging (Figure 3b) were performed to verify the accuracy of the canal filling. Finally, a permanent composite filling (Gradia Direct, GC, Tokyo, Japan) was placed. The patient returned for follow-up visits at 3 and 6 months, reporting a complete resolution of pain. Clinically, there was no pain upon vertical percussion, and there was no tenderness upon palpation of the alveolar process. The buccal mucosa presented without clinical changes. The mobility of the tooth was within the physiological limits (Grade 1 Miller mobility index) [15]. Radiographically, there were no signs of external resorption or inflammation around the tooth fragments and there were no periapical changes observed. The patient was advised to continue with regular follow-ups. At the 12-month follow-up visit, the patient exhibited no deviations from normal clinical findings. The examination revealed consistent results with the previous visit, showing no signs of discomfort, tenderness, or radiographic abnormalities (Figure 6 and Figure 7). The patient was informed about the need for further regular check-ups and to urgently report for a visit in case of any disturbing symptoms, such as recurrent pain, increased tooth mobility, or swelling.

## 3. Discussion

The results of this study emphasize the importance of using 3D-printed endodontic templates to improve the precision and effectiveness of endodontic treatment, especially in cases of root canal obliteration [21]. Depending on the degree of PCO, root canal treatment can be difficult even for experienced endodontists [22]. Effective localization of canal orifices using templates not only increases the probability of successful treatment but also minimizes the risk of iatrogenic complications [23,24]. Table 1 presents a comprehensive summary of the step-by-step procedure described in this document [11]. Krastl et al. [18] presented a digitally guided method for localization of obliterated canals, emphasizing the effectiveness of using endodontic templates, showing a significant reduction in treatment duration and minimizing the number of complications. Studies have shown that the use of such templates improves treatment results and helps in better preservation of tooth structure. Such an approach is part of conditional treatment, which is confirmed by the recommendations for the use of digital technology in endodontics [12]. Importantly, in this particular case, the use of guided endodontics was crucial to achieving a successful result. In our case report, without the precision provided by the 3D-printed template, conventional endodontic treatment would have been considered impossible and the tooth in such a young patient would have been considered for extraction. The successful outcome in this case, despite the presence of a double root fracture, challenges the commonly accepted belief that extraction is the only option for such teeth. This highlights the potential of guided endodontics to expand the possibilities of tooth preservation in complex cases. However, further studies are needed to assess the long-term success rates of endodontic treatment in teeth with complex root fractures. Clinicians should be aware of the need for continuous training and updating of knowledge on the use of digital technology. Nevertheless, despite promising results, further studies are needed to better understand the long-term effects and optimization of treatment protocols using endodontic templates. Future studies could include larger patient cohorts and different etiologies of PCO to establish the efficacy and safety of this novel technology in a wider range of clinical applications [25].

## 4. Conclusions

This study shows that the use of 3D-printed endodontic templates enables endodontic treatment in teeth with PCO, where treatment would otherwise be impossible. By providing precise access to the root canal system, these templates allow for optimal preservation of tooth tissues and improved prognosis. In the presented case, the use of endodontic navigation had a decisive impact on the preservation of the tooth, which would otherwise have been indicated for extraction. After a year of clinical and radiological follow-up, no deviations from the norm were found, and most importantly, the patient remains pain-free with preserved tooth function. The patient, a young 19-year-old, was able to avoid the psychological and functional consequences of tooth loss thanks to this innovative approach. This highlights the potential benefits of using this method in cases with complex etiology. However, in order to generalize the conclusions, it is necessary to conduct studies on a larger group of patients taking into account various clinical situations. Therefore, further research and dissemination of knowledge about templates are crucial to increase their availability and improve the standards of endodontics treatment.

## Figures and Tables

**Table 1:** Step-by-step digital and clinical process for guided endodontics.

CBCT scan and digital impression	- Intraoral: direct (scanning) or indirect (impression or plaster model). - Import DICOM and STL files into planning software.
Design and print endodontic guide	- Plan the drill path and create a guide Via 3D printing. - Verify fit before and after rubber dam placement. - Mark the access point on the guide for untreated teeth.
To access the canal	- Remove enamel until dentin is exposed. - Place and stabilize the guide on the teeth. - Use rotary burs through the guide to scout the canal in dentin. - Remove the guide, rinse the cavity, clean the burs, and verify access with a microscope.

## Data Availability

The original contributions presented in the study are included in the article. Further inquiries can be directed to the corresponding author.

## Abbreviations

The following abbreviations are used in this manuscript:

CBCT	Cone-Beam Computed Tomography
PCO	Pulp Canal Obliteration
Il-1	Interleukin-1
Il-6	Interleukin-6
TGF-β	Transforming grow factor-beta
TNF-α	Tumor necrosis factor-alpha
FGF	Fibroblast growth factor
FDI	Federal Dentaire Internationale
RVG	Radiovisiography
STL	Standard tessellation language
CWO	Continous Wave Obturation
PUI	Passive Ultrasonic Irrigation
